# Spontaneous fertility and variable spectrum of reproductive phenotype in a family with adult-onset X-linked adrenal insufficiency harboring a novel *DAX-1/NR0B1* mutation

**DOI:** 10.1186/s12902-020-0500-2

**Published:** 2020-02-06

**Authors:** Michelle Cerutti C. Vargas, Felipe Scipião Moura, Cecília P. Elias, Sara R. Carvalho, Nelson Rassi, Ilda S. Kunii, Magnus R. Dias-da-Silva, Flavia Amanda Costa-Barbosa

**Affiliations:** 1 Endocrinology Unit, Hospital Geral Alberto Rassi, Goiânia, Brazil; 2 Department of Medicine, Division of Endocrinology, Escola Paulista de Medicina, Laboratory of Molecular and Translational Endocrinology, Universidade Federal de São Paulo, Rua Pedro de Toledo 669, Sao Paulo, SP 04039-032 Brazil

**Keywords:** Adrenal hypoplasia congenita (AHC), Adrenal insufficiency, *DAX-1* mutation, Infertility

## Abstract

**Background:**

Adrenal hypoplasia congenita (AHC) is an X-linked disorder that affects the adrenal cortex and hypothalamus-pituitary-gonadal axis (HPG), leading to primary adrenocortical insufficiency (PAI) and hypogonadotropic hypogonadism. AHC is caused by a mutation in the *DAX-1* gene (*NR0B1*). More commonly, this disease is characterized by early-onset PAI, with symptoms in the first months of life. However, a less severe phenotype termed late-onset AHC has been described, as PAI signs and symptoms may begin in adolescence and adulthood. Here we describe a family report of a novel mutation within *NR0B1* gene and variable reproductive phenotypes, including spontaneous fertility, in a very late-onset X-linked AHC kindred.

**Case presentation:**

Three affected maternal male relatives had confirmed PAI diagnosis between 30 y and at late 64 y. The X-linked pattern has made the endocrinology team to AHC suspicion. Regarding the HPG axis, all males presented a distinct degree of testosterone deficiency and fertility phenotypes, varying from a variable degree of hypogonadism, oligoasthenoteratozoospermia to spontaneous fertility. Interestingly, the other five maternal male relatives unexpectedly died during early adulthood, most likely due to undiagnosed PAI/adrenal crisis as the probable cause of their premature deaths. Sequencing analysis of the *NR0B1* gene has shown a novel *NR0B1* mutation (p.Tyr378Cys) that segregated in three AHC family members.

**Conclusions:**

*NR0B1* p.Tyr378Cys segregates in an AHC family with a variable degree of adrenal and gonadal phenotypes, and its hemizygous trait explains the disease in affected family members. We recommend that *NR0B1* mutation carriers, even those that are allegedly asymptomatic, be carefully monitored while reinforcing education to prevent PAI and consider early sperm banking when spermatogenesis still viable.

## Background

Adrenal hypoplasia congenita (AHC) is an X-linked disorder that affects the adrenal cortex permanent zones and hypothalamus-pituitary-gonadal (HPG) axis, leading to primary adrenocortical insufficiency (PAI) and hypogonadotropic hypogonadism (HH) [1]. This rare condition is caused by a mutation in the *DAX-1*gene (dosage-sensitive sex reversal-AHC critical region on the X-chromosome 1), also called the Nuclear Receptor Subfamily 0 Group B Member (*NR0B1* gene) [1–3]. This gene is highly expressed in the developing urogenital ridge, pituitary, hypothalamus, gonads and adrenal cortex [4–6]. Classically, AHC with complete loss-of-function *NR0B1* mutations is characterized by early-onset PAI, with symptoms in the first months of life. However, a less severe phenotype termed late-onset AHC has been described, as PAI signs and symptoms may begin later in adolescence and/or adulthood [7–10]. Isolated mineralocorticoid deficiency can also be considered a milder phenotypic presentation [2, 11, 12].

In regards to pubertal aspects, most frequently, boys fail to enter puberty as a consequence of a combination of hypothalamic and/or gonadotropin pituitary dysfunction, resulting in permanent HH. In addition, infertility may result from primary testicular Sertoli cell injury [13] in a progressive fashion [14]. All these characteristics suggest that AHC is a highly variable disease.

Herein, we report a kindred with late-onset X-linked AHC harboring a novel *NR0B1* mutation, in which we have observed high variability of adrenal and gonadal manifestation, thus broadening the puzzling nature of AHC.

## Case presentation

**Fig. 1:**
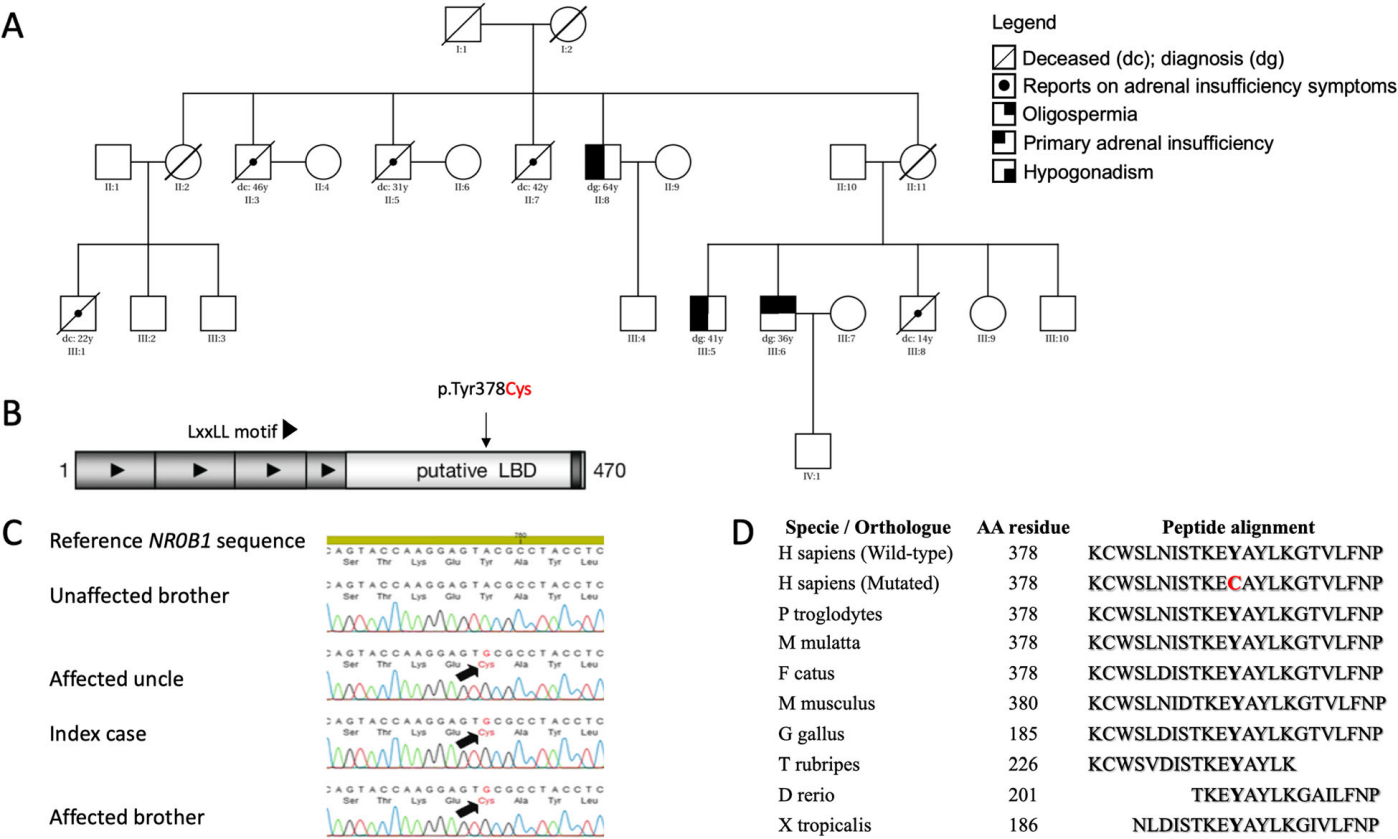
Molecular diagnosis of DAX-1/NR0B1 p.Tyr378Cys mutation segregating with adrenal hypoplasia congenita and variable degree of hypogonadism and infertility. **a** Pedigree of p.Tyr378Cys kindred depicting family members with primary adrenal insufficiency, hypogonadism and infertility. **b** DAX-1 peptide domain representation, which the arrow indicates the p.Tyr378Cys mutation in ligand binding domain (LBD) where other hotspot mutations causing adrenal hypoplasia congenita have been described. **c** Representative chromatogram is shown together with DAX-1/*NR0B1* gene reference sequence. The position of the mutation is indicated by a black arrow. **d** Peptide alignment among DAX-1/NR0B1 orthologues demonstrating its high conserved LBD region

**Fig. 2:**
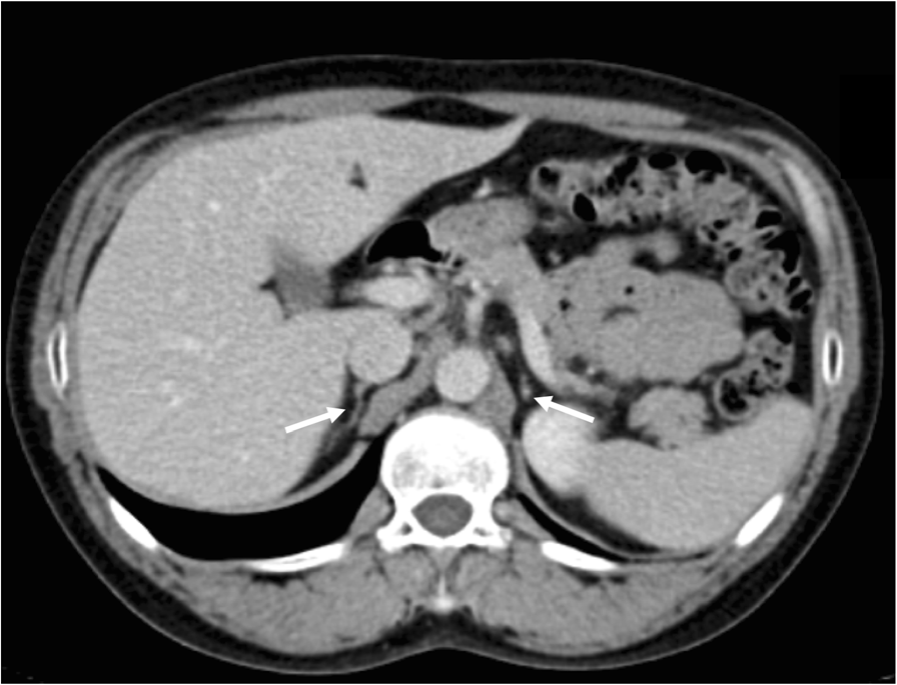
The contrast-enhanced computerized tomography scan demonstrating bilateral adrenal hypoplasia from the DAX-1 mutated index patient. White arrows indicate diminished adrenal glands from the case III.5

The index case is a male, 41 y (Fig. 1a: III.5), was admitted to the State General Hospital Emergency Department with an adrenal crisis due to irregular glucocorticoid and mineralocorticoid therapy for PAI. Since 30 y, he has been presenting with progressive weight loss, salt craving, and cutaneous hyperpigmentation. Although he allegedly had normal pubertal development, since he was 25 y, he has been complaining about erectile dysfunction and decreased libido. Physical examination was remarkable for cutaneous hyperpigmentation and decreased testicular volume (3 ml bilateral) and normal pubic hair distribution. Both height and span were 164 cm, body mass index (BMI) at 28 kg/m2. Laboratory results were compatible with PAI (Table 1). Adrenal antibodies were negative. HPG axis evaluation showed low total testosterone (TT) at 72 ng/dL, Follicle stimulating hormone (FSH) at 25 mUI/mL and Luteinizing hormone (LH) at 3.4 mUI/mL, and undetectable inhibin-B (see Table 1 for reference ranges). Computed Tomography (CT) revealed an important volume reduction in the adrenal glands (Fig. 2).

**Table 1:** Summary of the clinical and lab findings observed in p.Tyr378Cys Brazilian kindred presenting with very late-onset primary adrenocortical insufficiency and distinct reproductive phenotypes

Clinical and Laboratory Feature	Index Case	Affected Uncle	Affected brother
Age at PAI diagnosis (years)	40	64	36
Age at diagnosis of hypogonadism (years)	41	58	–
Cortisol (3–20 mcg/dL)	2.2	1.5	0.6
ACTH (<46 pg/mL)	1151	1012	>1250
Testosterone (220–715 ng/dL)	72	135	784
SHBG (11.2–78.1 nmol/L)	NA	NA	60
LH (0.6–12 mUI/mL)	3.4	13	3.3
FSH (0.9–12 mUI/mL)	25 (1.4–18)	24 (0.9–12)	13 (0.9–12)
Inhibin B (11–369 pg/mL)	< 4.8	< 4.8	63
Spermogram	NA	Azoospermia	Severe oligospermia, asthenospermia, teratospermia

*ACTH* Adrenocorticotropic hormone, *SHBG* Sex hormone biding globulin, *LH* Luteinizing hormone, *FSH* Follicle-stimulating hormone, *PAI* Primary Adrenal Insufficiency, *NA* Not available

Few months after index case PAI diagnosis, maternal index’s uncle (Fig. 1a: II.8), 64 y, presented at the Emergency Room (ER) at Countryside Hospital due to refractory hypotension. PAI diagnosis was then confirmed in the presence of hyperpigmentation, hyponatraemia, hypokalaemia, persistent nausea, low cortisol, and increased Adrenocorticotropic hormone (ACTH) levels (Table 1). Six years before PAI diagnosis was complaining about hypogonadism symptoms (e.g., low libido and erectile dysfunction), but he has declined any additional medical investigation. Physical examination at the ER demonstrated bilaterally reduced testicular volume (4 mL) and normal pubic hair. Height was 178 cm, span 177 cm, and BMI at 35 kg/m2. He fathered a healthy son at 39 y. Laboratory findings showed low TT at 135 ng/dL, FSH at 24 mUI/mL, and LH at 13 mUI/mL, plus undetectable inhibin-B and azoospermia (Table 1).

The proband’s brother (affected brother) (Fig. 1a: III.6) was diagnosed with PAI at 36 y, practically at the same time as his uncle. Dizziness, involuntary weight loss, hyperpigmentation, and fatigue were present since his early 30s. He has recently married and fathered a healthy son at 32 y. Height is 172 cm, span is 172 cm, and BMI is 21 kg/m^2^. The testicular volume was 12 mL bilaterally with normal pubic hair. Laboratory results have shown both normal TT (784 ng/dL) and inhibin-B levels (63 pg/mL), but severe oligoasthenoteratozoospermia was detected (Table 1). Interestingly, the other five male family members unexpectedly died in adolescence or adulthood between 14 and 46 y (Fig. 1a: II.3, II.5, II.7, III.1, III.8). Although no precise investigation was performed, family reports of adrenal insufficiency symptoms in these members led us to conclude that undiagnosed adrenal crisis was probably the cause of their early deaths.

Because of the PAI X-linked pattern in the family, *NR0B1* gene mutation associated late-onset AHC was strongly suspected. Sanger sequencing revealed a novel homozygous p.Tyr378Cys (c.1133A > G, cDNA 1368A > G, g.1368A > G) *NR0B1* mutation (Fig. 1b-c). This mutation segregated in all three PAI family members III-5, III-6, and II-8 but not in the healthy tested sibling (III.10). We tested several protein predicting bioinformatics algorithms including PolyPhen [15] (Polymorphism Phenotyping, http://genetics.bwh.harvard.edu, Harvard), PROVEAN [16] (Protein Variation Effect Analyzer, http://provean.jcvi.org/index.php, JCVI), MutationTaster [17] (MutationTaster, http://mutationtaster.org, NCB) and Have Our Protein Explained (HOPE) http://cmbi.ru.nl/hope, CMBI) [18]. All four in silico analysis algorithms were concordant to predict that tyrosine to cysteine amino acid substitution can cause disruption of DAX-1 function. Tyr378 residue is part of a highly conserved sequence motif across orthologous sequences (Fig. 1d).

## Discussion and conclusion

We describe a late-onset X-linked AHC family harboring a novel *NR0B1* mutation that segregated in three affected family members. Interestingly, these individuals present remarkably variable gonadal features that may add more insights to the heterogeneity of the disease. Although early-onset PAI and pubertal development defects are the most common phenotypic features of X-linked AHC [1], its diagnosis in adulthood, also called the late-onset form, had been recognized [7–10, 14, 19–21]. AHC phenotypic variability has been described even among patients carrying the same *NR0B1* mutations [1, 22].

Although the precise PAI onset has been difficult to determine, all affected males (including the deceased ones) in the reported family may have had symptoms during adolescence up to 64 y. In agreement with Kyrikiakis et al. [7], there is no rule regarding the temporal pattern between hypogonadism and PAI onset. Our family presented hypogonadism symptoms preceding PAI in the index case and his uncle but not in the affected brother. Additionally, there was no history of overt PAI symptoms or delayed puberty in alive family members, including in female one (III.9). Indeed, some female carriers of DAX1 mutation have been reported presenting with PAI or puberty delay [23, 24]. These intriguing clinical manifestation in female carriers may reflect variable gene expression or even oligogenicity, similarly to other X- linked diseases [25].

It is well recognized that in addition to adrenal glands, *NR0B1* mutations disturb hypothalamic, pituitary or even gonad levels [1]. HH is the most common finding [1, 24]. However, delayed or incomplete puberty associated with partial gonadotropin deficiency [1, 26, 27] or phenotypes with normal gonadotropin function have also been described [12, 14, 28–31]. More puzzling, some have suggested that chronic ACTH stimulus to Leydig cells may be related to gonadotropin-independent precocious puberty phenotype in X-linked AHC boys [28]. The following HPG features of the three affected members deserve attention: a) PAI accompanied by isolated oligoasthenoteratozoospermia in affected brother (III.6), b) uncle and the index case (II.8 and III.5, respectively) have PAI and hypogonadism, both in the hypergonadotropic range, and c) proband’s uncle and affected brother (II.8 and III.6, respectively) fathered before X-linked AHC diagnosis. Perhaps, nuclear receptor steroidogenic factor 1 (*SF-1*) and many other unknown genes or epigenetic factors might interact with DAX-1 at different levels of the HPG axis, [3, 32, 33] favoring those assorted reproductive phenotypes.

Mouse models [34] reinforced by human findings [4, 14] have shown that *NR0B1* disruption can cause progressive degeneration of germinative cells, seminiferous tubules, and Leydig cells, resulting in sterility [34] and poor reproductive prognosis [8, 20, 24, 35, 36] despite normal serum testosterone and gonadotropins. Frapsauce et al. [13] reported the unique case of paternity after testicular sperm extraction/ intracytoplasmic sperm injection (TESE-ICSI) in a patient with *NROB1* mutation. Although the affected brother presented a significant reproductive phenotype “oligoasthenoteratozoospermia”, he still has preserved fertility, normal testosterone, and inhibin-B levels, contradicting those animal models. Oligospermia has been previously described in individuals with an *NR0B1/DAX-1* mutation [14]. In addition, Mou et al. [37] have described that p.V385 L mutation is associated to secretory azoospermia in individuals with no history of X-linked AHC [37]. Then we can speculate that ongoing testicular failure of the maternal uncle and isolated spermatogenesis defects in the affected brother, both with spontaneous fertility, may represent different steps of a DAX-1 progressive gonadal defect during their lifetime [14, 38].

**Fig. 3:**
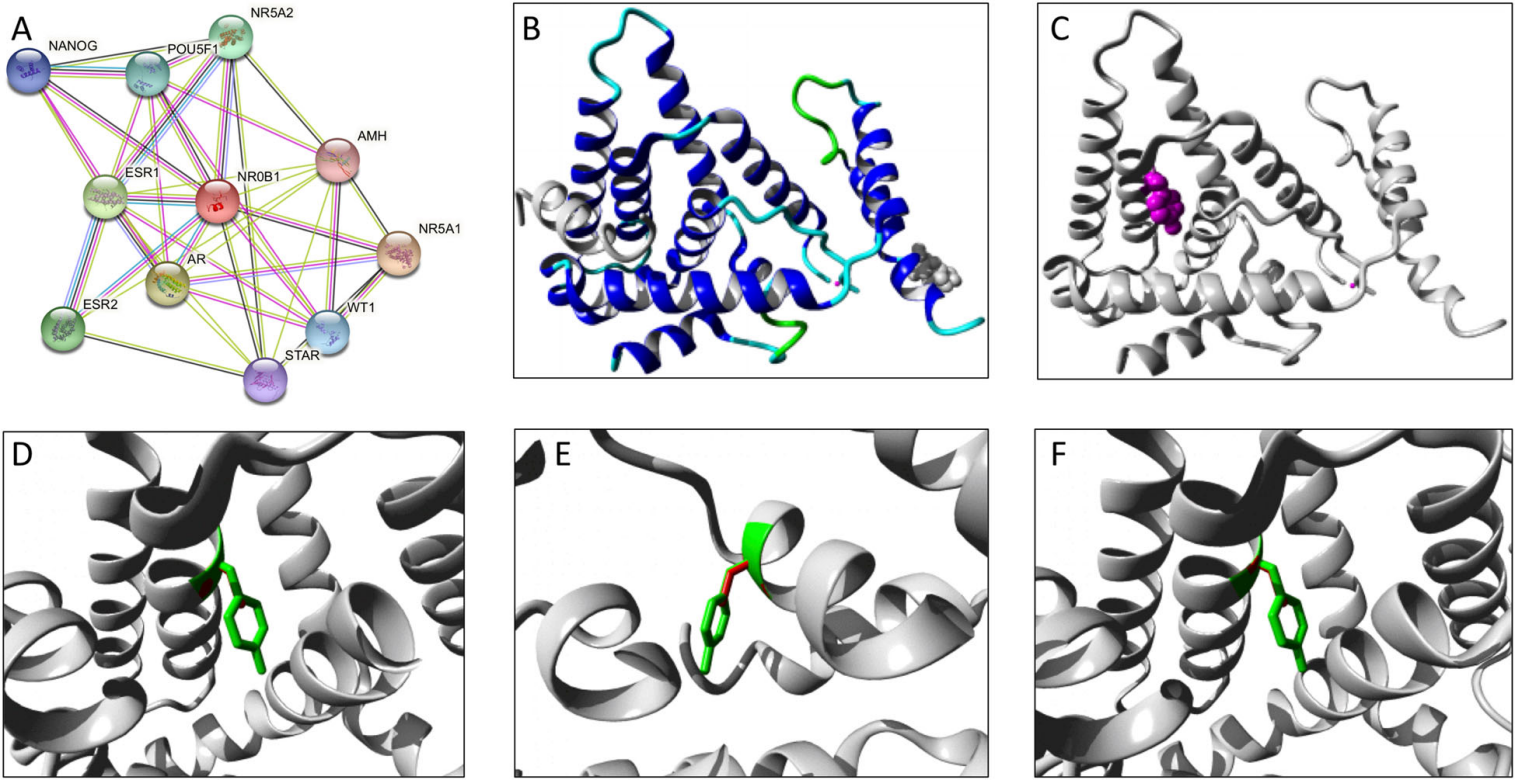
DAX-1/NR0B1 p.Tyr378Cys mutation in silico functional analysis. **a** Nuclear receptor subfamily 0 group B member 1 (NR0B1) is an orphan nuclear receptor that plays a critical role in the cascade required for the development of the hypothalamic-pituitary-adrenal-gonadal axis. DAX-1/NROB1 acts as a coregulatory protein on transcriptional activity of other nuclear receptors such as NR5A1, AR, ESR1, ESR2, NR5A2, POU5F1, WT1, NANOG, and STAR through heterodimeric interactions. These receptors are predicted as NR0B1 functional partners by using String software (https://string-db.org/cgi/). **b** Overview of a NR0B1 homologous protein structure in ribbon presentation, in which a-helix, ß-strand, turn, random coil and other interacting molecules (such as transcriptional factors) are highlighted in blue, red, green, cyan and grey respectively. **c** Ribbon NR0B1 presentation highlighting the side chain (small balls) of the mutated residue Cys378 in magenta. **d**, **e**, and **f** different close-ups of the side chains of both the wild-type (green) and the mutant (red) residue. These D, E and F shot evidence that the wild-type and mutant amino acids differ in size, which may result in an empty space in the core of the protein, besides its change in charge and hydrophobicity valu using Hope Version 1.1.1 software [18]

Although no in vitro functional studies were performed in p.Tyr378Cys, this highly conserved mutation (Fig. 1d) segregated in all affected individuals but not in the healthy relatives. Indeed, the in silico analysis was also considered disruptive. Even though the exact 3D-structure of DAX-1 is unknown, we were able to build a model based on a homologous protein structure using HOPE software (Fig. 3). The wild-type and mutant amino acids differ in size, being the mutant residue smaller than the wild-type residue. This change can result in an empty space in the core of the protein (Fig. 3c) as zoomed-in Fig. 3d-f. The hydrophobicity of the wild-type and mutant residue also differs and can cause loss of hydrogen bonds and as a result disturb correct protein folding, likely making difficult the heterodimeric interaction between DAX-1 and other coregulatory nuclear receptors such as NR5A1, AR, ESR1, ESR2, NR5A2, POU5F1, WT1, NANOG, and STAR (https://string-db.org/cgi/) (Fig. 3a).

The mutation p. Tyr378Cys is close to the previously described late-onset AHC mutation p.Tyr380Asp [8], in which transient gene transcription assays resulted in partial loss of DAX-1 function. Additionally, this mutation and the other described late-onset AHC mutations (p.Ser259Pro, p.Pro279Leu, p.Tyr380Asp, p.IsoI439Ser) are located within the putative carboxyl ligand-binding domain (LBD) [7, 14, 21]. These data suggest that mutations in the LBD region are less severe.

In summary, we described a novel *NR0B1*mutation in a late-onset AHC family with variable hypogonadal and reproductive features, including spontaneous fertility in two members. The very late PAI diagnosis (64 y) indicates that there may be other unknown factors responsible for this singular outcome. Our findings may bring more awareness when counseling allegedly asymptomatic AHC family members. Clinical evaluation throughout life with periodical exams, including spermogram and early sperm banks, should be performed may warrant fertility preservation on AHC males and diagnose very late PAI, therefore, improving patient quality and expectancy of life.

## Data Availability

The data that support the findings of this case report are available from the corresponding author on request.

## Abbreviations

ACTH: Adrenocorticotropic hormone
AHC: Adrenal hypoplasia congenita
BMI: Body Mass Index
CT: Computed Tomography
*DAX-1*: Dosage-sensitive sex reversal - AHC critical region on the X-chromosome 1
DNA: Deoxyribonucleic acid
ER: Emergency room
FSH: Follicle stimulating hormone
HH: Hypogonadotropic hypogonadism
HPG: Hypothalamus-pituitary-gonadal
LBD: Ligand binding domain
LH: Luteinizing hormone
NA: Not Available
*NR0B1*: Nuclear receptor subfamily 0 group B member 1
PAI: Primary adrenocortical insufficiency
SF-1: Steroidogenic factor 1
TESE-ICSI: Testicular sperm extraction - Intracytoplasmic sperm injection
TT: Total testosterone
